# Coordinated response of endemic gastropods to Late Glacial and Holocene climate-driven paleohydrological changes in a small thermal pond of Central Europe

**DOI:** 10.1038/s41598-024-60185-5

**Published:** 2024-04-24

**Authors:** Sándor Gulyás, Pál Sümegi

**Affiliations:** Department of Geology and Paleontology, University of Szeged, Szeged, Hungary

**Keywords:** Evolution, Palaeontology, Limnology

## Abstract

The thermal spring-fed Lake Pețea located in NW Romania southeast of the city of Oradea harbors a unique endemic warm water biota. It is the only location in Europe where thermal water endemic melanopsid *Microcolpia parreyssii* (Philippi, 1847) lived along with the highly endangered warm-water relict neritid *Theodoxus prevostianus*. Lake Petea’s evolution was mainly controlled by major climate-driven hydrological changes also seen in regional records. The hydrological changes were mainly controlled by varying input of thermal water due to recurring increased/decreased recharge of the underground karst water system. The driving factor was warming connected to the interstadial GI 1 increasing recharge by melting of regional ice sheets in the Late Glacial. Conversely, during the Younger Dryas (H0) and the Holocene increasing/decreasing moisture availability was in control. Low stands created multiple bottlenecks reducing genetic variability seen in the appearance of extreme morphologies during next rapid climate melioration. The studied gastropods responded mostly similarly to changes controlling the availability of elements in shell construction and habitat reduction leading to changes in shape, density, size. Periods of lower lake levels and reduced warm water input are characterized by the emergence of elongated tightly coiled shells while globular, compressed loosely coiled shells develop at times of warmer water provision and increased Mg availability. In size there is a contrasting trend. Namely globose *Th. prevostianus* shells are larger than the elongated ones. Conversely globose, compressed *Microcolpia* are generally smaller than their elongated spindle-shaped counterparts. In this sense the development of dwarf morphotypes in warmer water habitats is characteristic of Lake Pețea melanopsids. This type of dwarfism i.e. the reduction of shell size is lacking though in Lake Pețea neritids. Our findings also confirm the presence of various ecophenotypes of *Microcolpia* in the pond degrading our endemic species *Mi. parreyssii* to a variety of *Mi. daudebartii*.

## Introduction

Intraspecific shell variation is common in freshwater gastropods, the mechanism that give rise to this variation however are often unclear^1–7^. One source of shell variation is phenotypic plasticity, when a single genotype gives rise to multiple phenotypes through developmental responses to biotic or abiotic environmental factors such as presence of predators, stream flow, oxygen and Ca^2+^ limitation, habitat reduction, water chemistry and temperature changes etc.^7–11^. For testing the hypothesis of phenotypic plasticity evoking shell shape variation, the most common approach is the careful documentation of shell related traits followed by an evaluation of its spatial, temporal variance considering recorded or inferred environmental parameters.

Understanding the underlying reasons for phenotypic plasticity and the resulting morphological disparity is thus one of the key topics of evolutionary biological and paleobiological research on mollusks^1–3,5–11^ The phenotypic plasticity of extant and fossil melanopsids (the family Melanopsidae H. & A. Adams, 1855) has been widely documented in various lacustrine and fluvial habitats^12–27^. However, studies of small thermal water habitats harboring highly endangered endemics and with geological records spanning several millennia is rare^28^.

Thermal ponds represent unique habitats with a special temperature regime and chemical composition^28^. Temperatures are higher than in other habitats and can fluctuate frequently. The chemical composition is likewise variable depending on the bedrock, thermal water source and composition etc. Ecological systems in isolated small range thermal spots are the most imperiled because of frequent fluctuations in thermal water supply and consequent fluctuations in water temperature, availability of elements for shell construction and metabolic processes and lake levels as well as trophic status due to natural processes and/or increased pressure from humans to use geothermal resources. On the other hand, these sudden rapid or slow changes in the environment may favor the multiple iterative emergences of different phenotypes of the same genotype.

Within the gastropod family Melanopsidae members of the genus *Microcolpia* are restricted to small habitats of thermal springs and spring-fed ponds of Central and Central Eastern Europe^29^. Classical examples are *Microcolpia daudebartii daudebartii* (Prevost, 1821) from Bad Vöslau and Bad Fischau, Austria^26,27,30–32^, *Microcolpia daudebartii thermalis* (Brot, 1868) and *Microcolpia daudebartii acicularis* from the Bükk Mts. NE Hungary^33–35^, as well as thermal creeks of NW Romania near Rabagani^36,37^.

**Figure 1:**
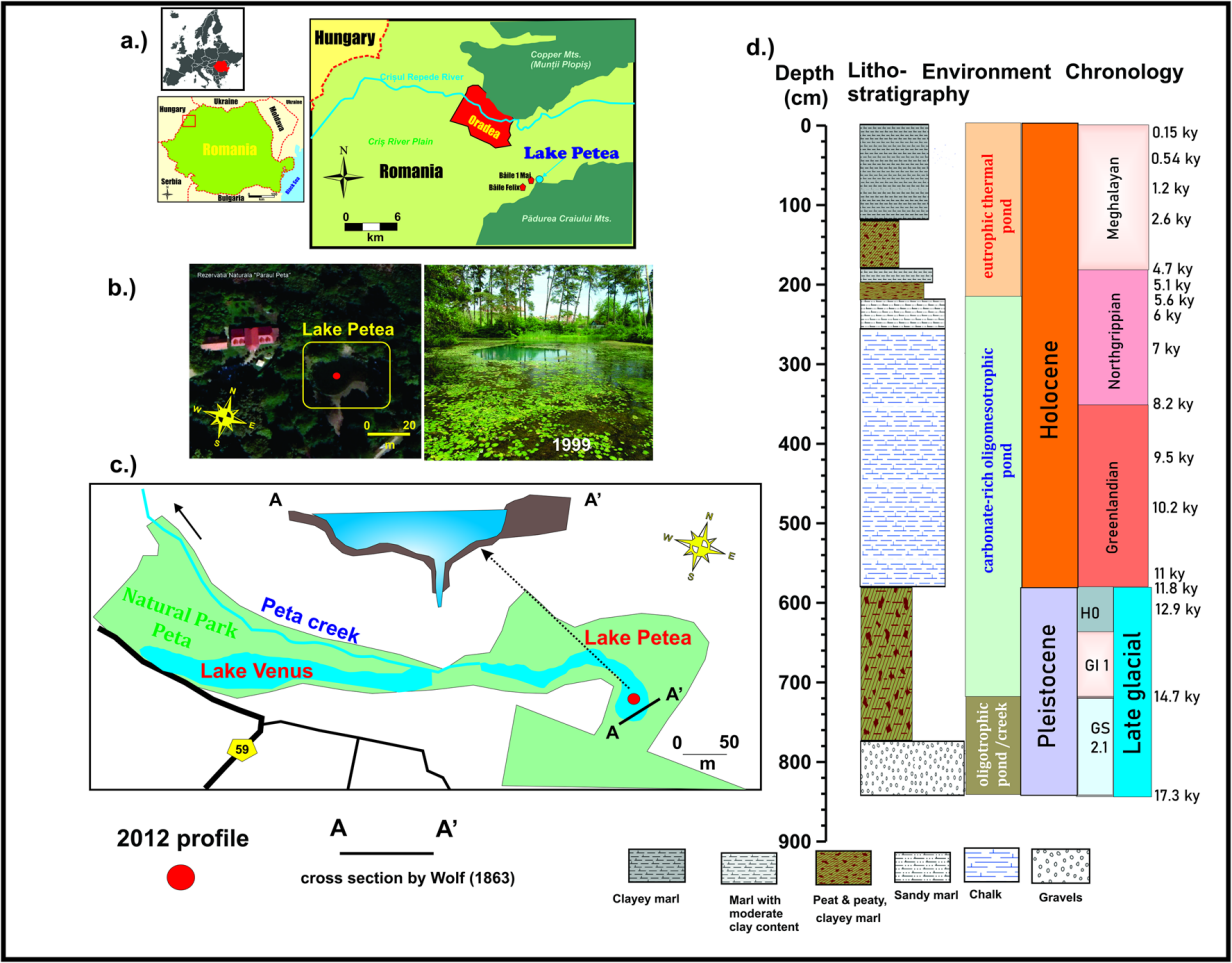
Location (**a**), environment (**b**,**c**) and litho-, chronostratigraphy of the studied thermal Lake Pețea sequence (**d**) (photograph taken by P. Sümegi, maps were created from Google Maps and Google Earth using the software Coreldraw 2021).

The thermal spring-fed Lake Pețea located in NW Romania southeast of the city of Oradea has a unique endemic warm water biota. It is the only location in Europe where the now extinct thermal water endemic melanopsid *Microcolpia parreyssii* (Philippi, 1847) lived together with the also extinct endemic neritid *Theodoxus prevostianus*^26,38–48^. Lake Pețea drained by the Peța creek is a very shallow lake with a maximum depth of 2–3 m and an area of a couple of tens of square meters, with average water temperatures of 30 °C^37,42,44,49–51^ (Fig. 1). The bicarbonate, calcium, and sulfate-rich waters of the lake are maintained by underwater thermal springs deriving from the Lower Cretaceous aquifer system^50,52–54^, so fluctuations in spring activity is the major driver of lake level changes. Thus, it is very prone to natural and artificial environmental changes, i.e. fluctuations in depth, the surface area along with water temperature, chemistry, nutrient supply, and substrate conditions leading to habitat and food source loss and concomitant increased predation^42,49,50,55,56^.

**Figure 2:**
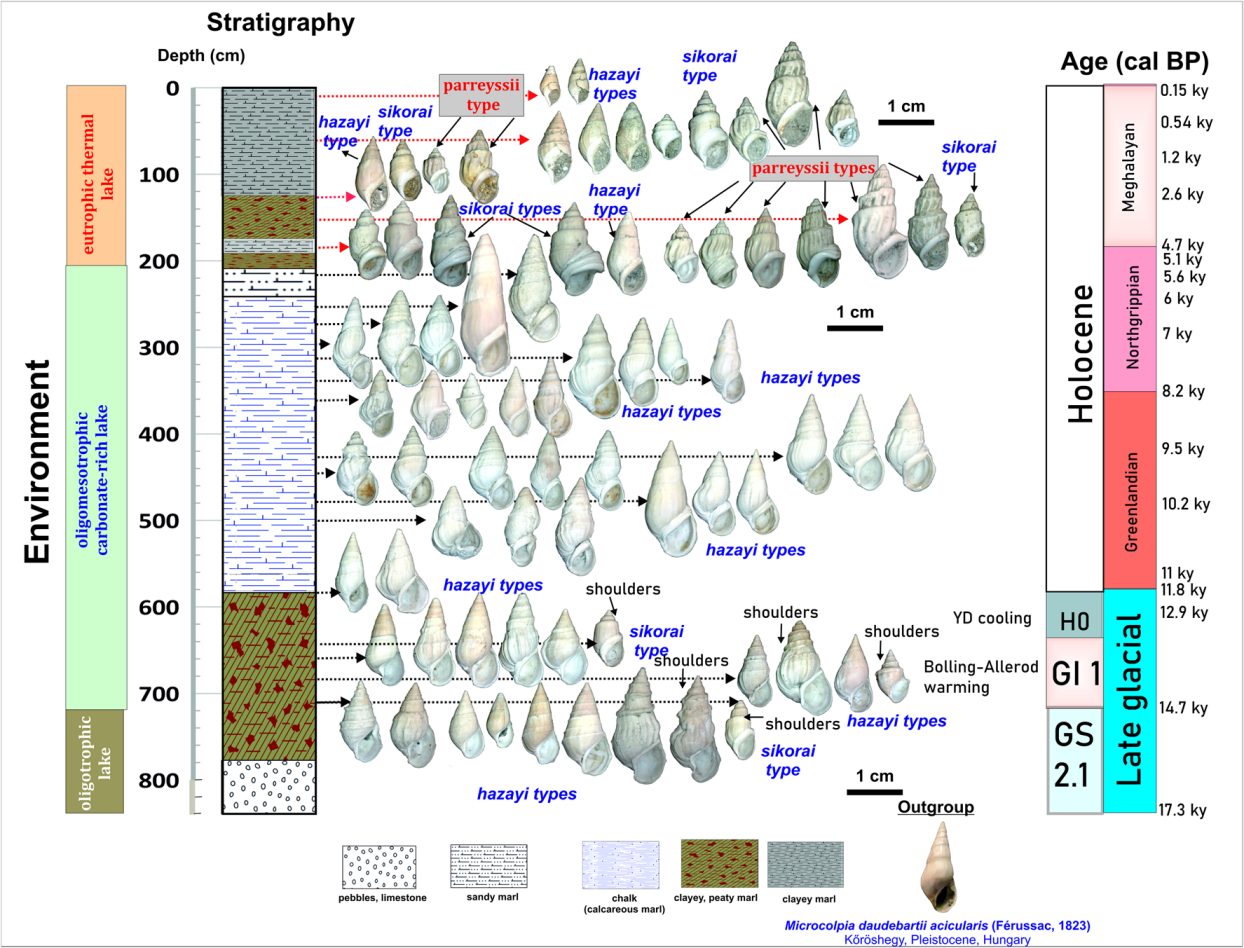
Litho-, chronostratigraphy of the studied Lake Pețea sequence with the temporal occurrence of the most prominent endemic *Microcolpia* types (note the early appearance of shouldered morphotypes during GI 1 of the Late Glacial).

Extant phenotypes of *Microcolpia parreyssii* have well-defined morphological traits of pronounced shouldering and the overall presence of axial ribs with notches along the suture. However, Holocene, and Pleistocene subfossil melanopsids show extraordinary morphological variation, from smooth, slender elongate, keeled specimens to bulkier smooth, ribbed specimens with varying degrees of shouldering, ornamentation, callus thickness, aperture shapes, and sizes (Fig. 2), which captured the attention of paleontologists and biologists very early and led to the description of over 40 species and types^26,35–41,43,45–48,57^.

Genetic studies however, pointed to a close genetic relationship of the mentioned *Microcolpia* taxa with minimal distances^29,57^ bringing up the possibility that both the extant and subfossil phenotypes correspond to the varieties of the ancestor *Microcolpia daudebartii acicularis.* A recent morphometric study of subfossil shells from known stratigraphic positions indicated the presence of two major groups (taxa) characterized by the different shapes of bulky, shouldered shells and elongate spindle-shaped shells^58^. The former were considered phenotypes of *Microcolpia parreyssii.* The latter was considered phenotypes of *Microcolpia daudebartii hazayi*^58^*.* Yet the exact taxonomic status of the subfossil taxa remains unresolved. Evidence of the presence of ecophenotypes through a close temporal relationship between shape and environmental parameters can clarify the shape variation caused by environmental stimuli. Understanding how shell characteristics have changed over time given the changing environment, can also help identify possible drivers of morphological differences.

Temporal variations of morphological traits in endemic melanopsids and neritids of known stratigraphic position have yet to be presented. Thus, a reliable understanding of factors driving morphological evolution is lacking. So, the major aims of the present paper are (1) provide a high-resolution paleohydrological reconstruction of the lake via complementing previously published sedimentological, palaeoecological results^54^ with geochemical, isotope geochemical analysis of lacustrine sediments and melanopsid, neritid gastropod shells (2) reconstruct the temporal variation of morphological disparity and reveal potential underlying causes relying on the newly established paleohydrological, biogeochemical data. (3) compare morphometric and isotope geochemical data with rescued specimens from Lake Petea presented by Müller et al.^42,59^ to understand if and how the morphology of *Mi. parreyssii parreyssii* changed in the modern lake leading to the preservation of a single taxon from the former wide array of morphotypes.

## Results

### Reconstructed paleohydrology of the lake

**Figure 3:**
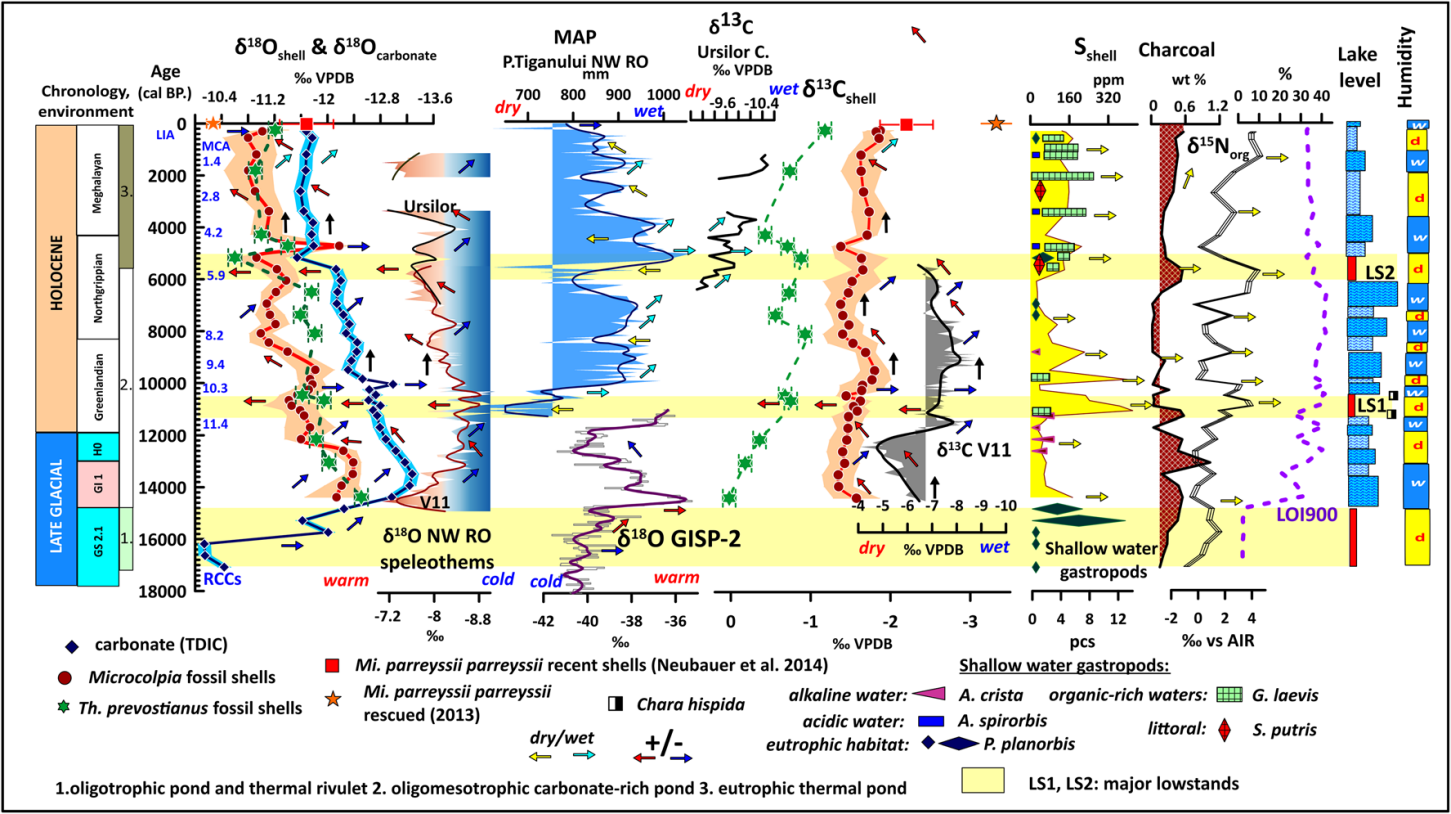
Comparison of geochemical data of shells, lacustrine carbonates, organic matter, charcoal concentrations, abundance of shallow water gastropods with regional speleothem, precipitation proxies, and isotope data for GISP-2.

**Figure 4:**
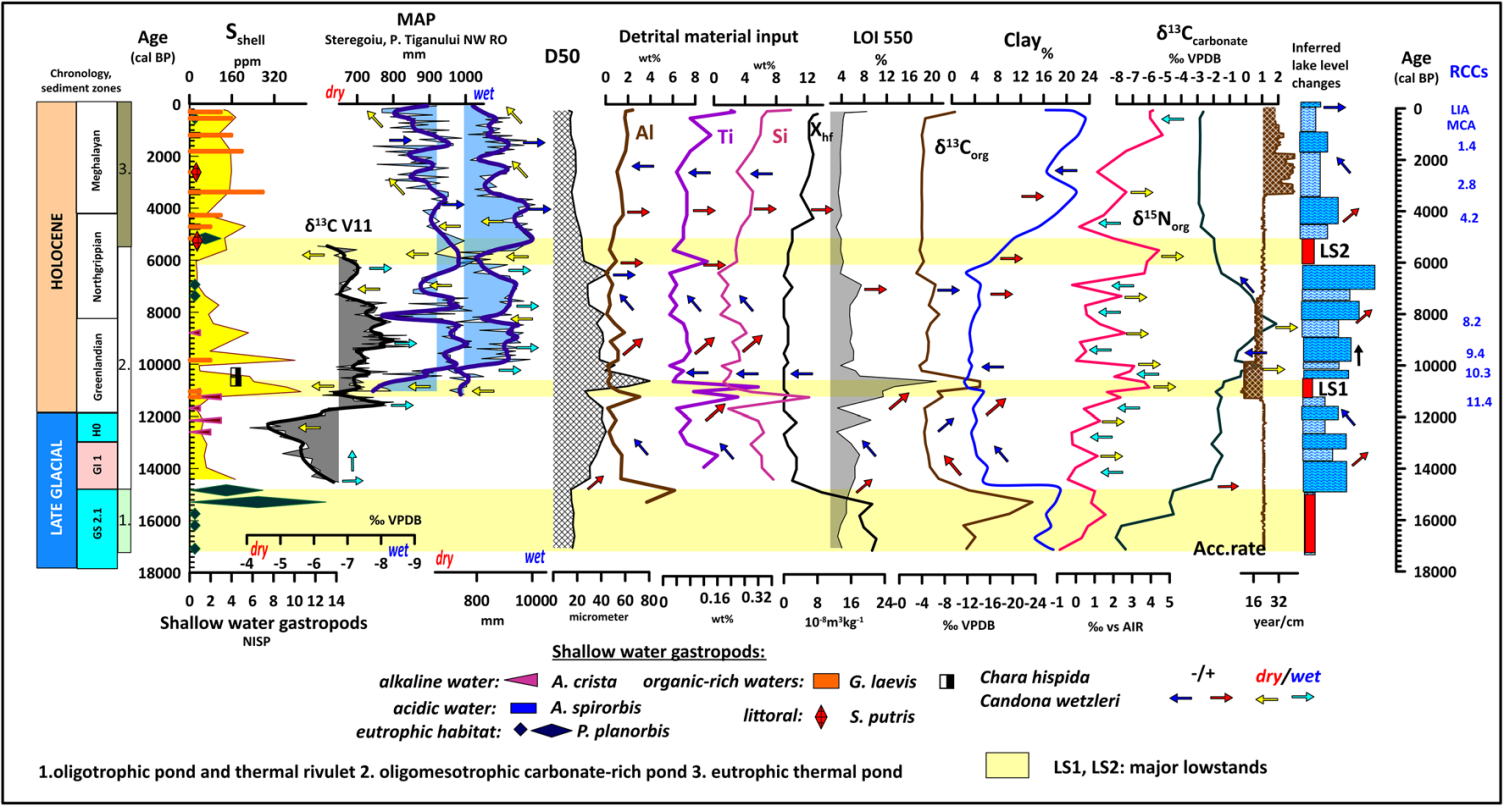
Comparison of variations in geochemical data of shells, organic matter, grain-size and sedimentation rates, detrital input, abundance of shallow water gastropods with regional speleothem, precipitation proxies, inferred lake level changes.

The initial phase of the lake’s evolution corresponds to the period from 17 ka cal BP to 14.7 ka cal BP.; i.e., the Oldest Dryas. The age of thermal waters in the Cretaceous aquifer is around 20.3 ka cal BP^52^. So, an initial charge-up of the system must have started shortly after the nadir of the LGM and must have reached the top part of the reservoir by the Late Glacial. In this part, a shallow oligotrophic lake was formed with a considerable silt input and minimal clay and carbonate content in its sediments (Figs. 3, 4). The presence of limestone pebbles at the base of the sequence hints at the activity of a rivulet in creating the lake basin. Shells were highly fragmented or lacking in this interval. Initial δ^18^O_carbonate_ values (− 10.3‰) are close to the modern precipitation (− 9.9‰)^60^ (Fig. 3). δ^13^C_carbonate_ values are low (− 6 to − 8‰) close to characteristic values of river waters recorded in W. Europe (− 6 to − 10‰)^61–63^ implying that at this stage the only source of water was reduced precipitation and riverine water input in line with the observed presence of carbonate pebbles (Fig. 3). Based on our results the dominant minerals are calcite, quartz, and muscovite present in the other parts of the sequence too. However, it is the only zone where clay minerals of illite and kaolinite appear marking increased physical and chemical weathering around the lake basin (SFig 1). Elements of detrital origin (Al, Ti, Si) have the highest values (20–25 wt%), in addition to magnetic susceptibility (18 * 10^−8^ m^3^ kg^−1^) and clay content (17%) confirming the input of significant amounts of magnetic mineral-rich sediment into the basin at times of higher rivulet discharge (Fig. 4). Highly negative δ^13^C_org_ values (− 12 to − 24‰) confirm increased bioproduction and lower water levels in relatively drier conditions shown by upwards increasing δ^15^N values (0–2‰), as organic matter produced by algae thriving in deeper waters is depleted in δ^15^N (Figs. 3, 4). The lack of carbonate nodules and the minimal carbonate content (2–3%) indicates a complete choking of the conduit of the thermal water spring feeding the lake. Sedimentation times are high indicating slow accumulation (22–24 years/cm) (Fig. 4). From the aquatic gastropods the shallow eutrophic water preferring *Planorbis planorbis* is present. Shells are minimal and highly fragmented.

There is a stepwise change in all parameters around 14.7 ka cal BP (Figs. 3, 4). The stepwise increase in carbonate concentration (to 23.4%), average grain size (30 µm), and Ca, Mg with a parallel significant reduction of the clay content (to 4%) and increase in the organic content (to 8%) marks the next phase of lacustrine evolution into an oligomesotrophic carbonate-rich lake.

Wohlfarth et al.^64^ from Preluca Tiganului, NW Romania, obtained a similar age for the beginning of lake sedimentation. δ^13^C_carbonate_ values are positively shifted to values ranging between − 3 and + 2 in the remaining part of the sequence. These are characteristic of karstic water systems enriched in δ^13^C within the mentioned range because of carbonate dissolution^61–63^ marking the first major input of underground thermal waters. The marked negative shift in oxygen isotope values of both shell and sediment carbonate to all-time lows (Fig. 3) close to marginal values of the Triassic thermal waters (− 12.3‰)^52,60^ clearly signals the increased input of deep-sourced thermal waters depleted in oxygen due to meltwaters from glaciers. A parallel positive shift of δ^13^C_shell_ is also notable owing to the dilution of the water. It is also the period when the first representatives of the thermophilus melanopsids and neritids appear in the sequence marking the development of relatively warmer waters with temperatures around 16 °C^45–47^ most likely because of the significant warm water input. This transition is coeval with the Bølling/Allerød interstadial (GI-1). The beginning of GI-1 in the Greenland Ice Core shows a rapid and considerable temperature amplitude increase of 10 °C^65,66^ (Fig. 3). The δ^18^O values of speleothems from NW Romania^67^ and the growth intervals of speleothem in Scărişoara Ice Cave^68^ also indicate a rapid warming phase initiating at ∼14.8 ka cal BP in the area (Figs. 3, 4).

**Figure 5:**
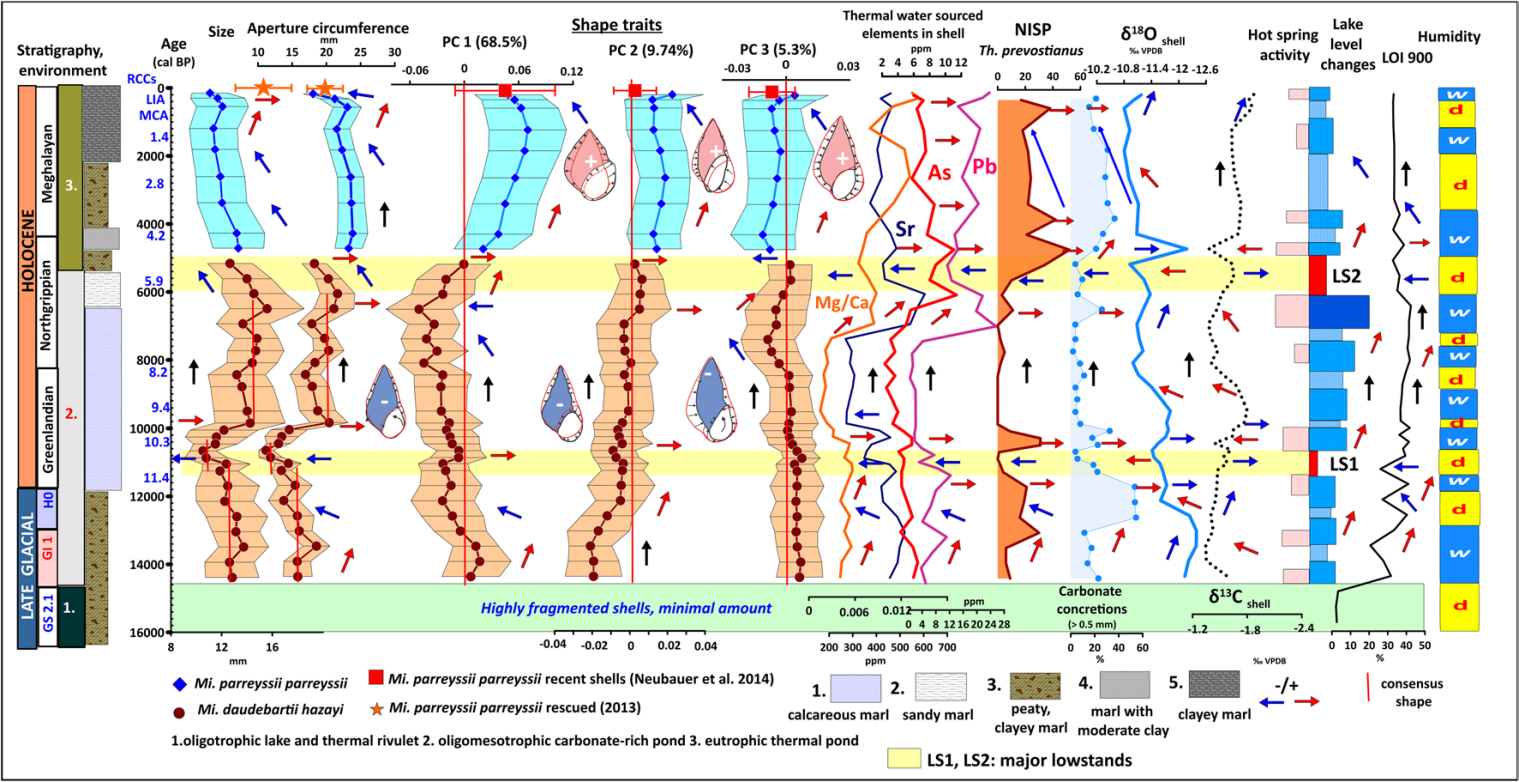
Temporal variation in shape and size parameters of fossil and extant *Microcolpia* in light of selected paleohydrological proxy data.

**Figure 6:**
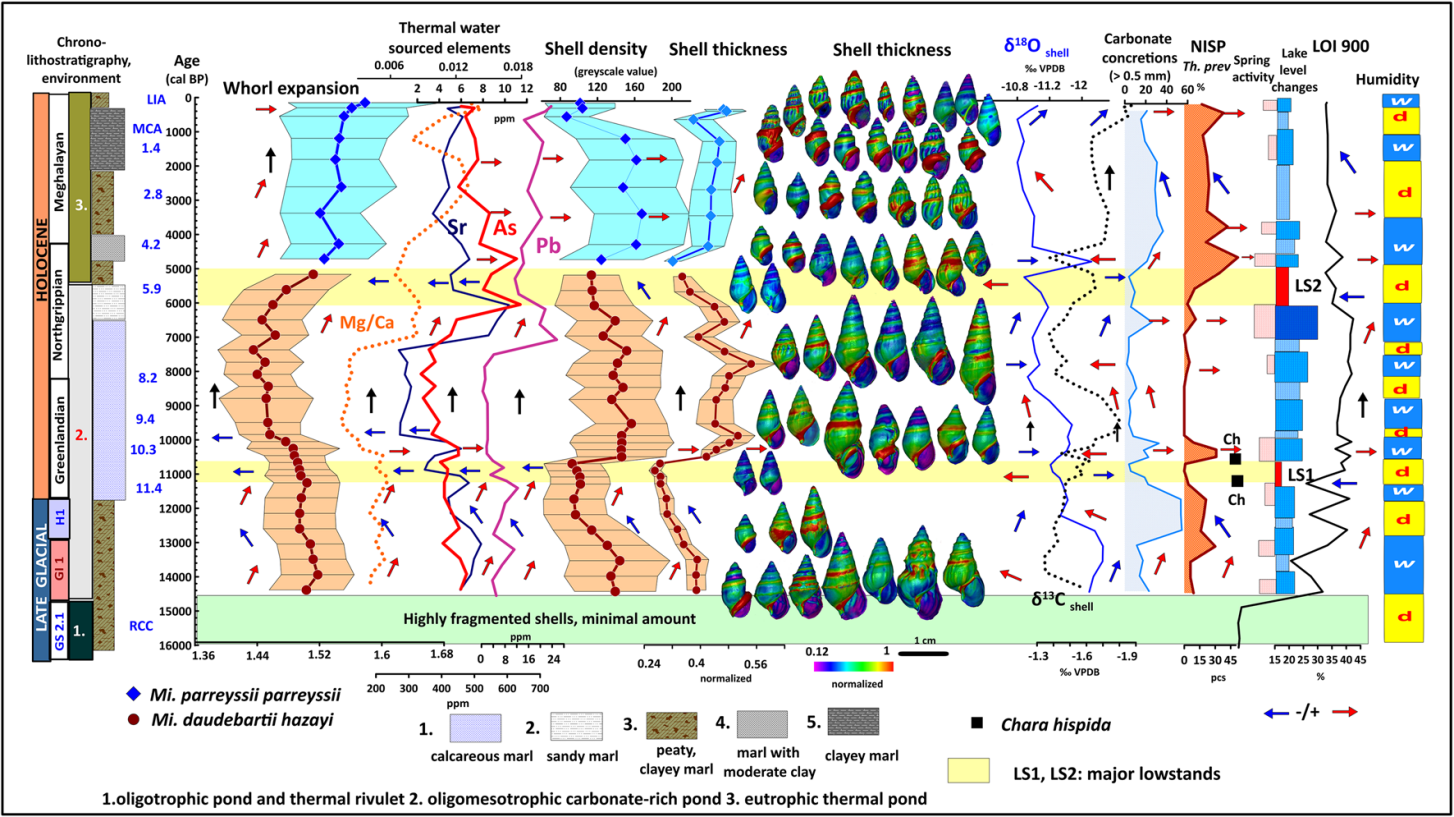
Temporal variation in shell density, whorl expansion and shell thickness of the two major *Microcolpia* taxa in light of paleoenvironmental data.

This warming trend is notable throughout Central Eastern Europe^68^. Coevally, increasing annual and summer temperatures are also seen in other pollen records from the region of NW Romania^69–71^ and chironomid-based reconstruction of summer temperatures in the South Romanian Carpathians indicating an increase of ∼2.8 °C in summer air temperature during the same transition^72^. The emergence of warmer humid conditions must have resulted in the melting of local icefields, increased precipitation, and an elevated recharge of the groundwater system resulting in higher discharge of the thermal springs in the area supplying warm waters to the lake. The sudden increase in the concentration of carbonate concretions and steady input of coarser grain fractions signals the reopening of the conduit of the underlying spring. It is also the time when the first peaks of thermal water sourced As, Sr, Pb appear in shells (Figs. 5, 6). The concentration of elements marking detrital material input decreases (Al, Si, Ti), a strong positive shift of δ^13^C_org_ values and negative shift of δ^15^N in addition to the disappearance of shallow water gastropods signals a deepening of the lake (Figs. 3, 4).

In the interval dated to the Younger Dryas (HO) an increase in charcoal concentrations in our lake marks dry conditions corroborated by positive δ^13^C values of nearby speleothems leading to lowered lake levels signaled by the appearance of shallow water gastropod taxa (Fig. 3). The δ^18^O values of speleothems from NW Romania^67,73^ is negatively shifted signaling cooling, while our shells and lake carbonate show a positive shift along with a decrease in thermal water sourced As, Pb and Sr in the shells (Figs. 5, 6) and the abundance of the thermophilus gastropod *Th. prevostianus*. δ^13^C_carbonate_ turns negative also signaling a reduced spring activity and the supply of δ^18^O depleted thermal waters due to a drop in the groundwater levels (Figs. 3, 4, 5, 6).

The next marked change is dated between 11.7 and 11.4 ka cal BP with a slight negative shift in our shell oxygen isotope values, a positive shift in shell carbon isotope values (Fig. 3). δ^13^C_carbonate_ turns back, a second peak of thermal water sourced As, Pb and Sr is notable after the one at GI-1, with an increase in small carbonate concretions and the abundance of the thermophilus *Th. prevostianus* marking renewed spring activity (Figs. 4, 5, 6). This must have resulted in a slight increase in the lake level. This period coincides with a rapid increase in precipitation connected to the 11.4 ka event also recorded in the negative shift of carbon isotope values of speleothems from NW Romania^67,73^ (Fig. 3). This rapid precipitation increases not only resupplied the underground springs with water but also increased nearby fluvial activity and erosional material transport into the already shallow lake as marked by the highest peaks of detrital elements (Al, Si, Ti), towards the end of the period (Fig. 4). There is a stepwise change in sedimentation to higher accumulation rates (8–10 y/cm) marking increased sediment input resulting in a rapid shallowing of the lake leading to the emergence of a marked low stand, one of the largest in the lake’s history (LS1) between 11.4 and 10.4 ka cal BP corroborated by the peak of organic-rich shallow water preferring gastropods, the highest peak in shell sulfur (460 ppm) indicating intensive swampification at a time when according to regional paleoclimatic and paleohydrological data marked dry conditions develop (Fig. 4). Early Holocene records for the lowlands of NW Romania show high summer temperatures (by ca. 4 °C) and ca. 33% lower precipitation rates^69,70^. High fire activity and a lowering of the lake level at Ştiucii Lake, and lower MAP inferred from the pollen records of the crater lakes of Steregoiu and P. Tiganului in NW Romania^71,74^ also indicate decreasing moisture availability in this interval*.* Speleothem records both in the southern and northern parts of the region^67,73^ from ~ 11.5 ka into the Holocene indicate a gradual warming trend (Figs. 3, 4). Regional records also show a decrease in lake levels as well as peat surface moisture in NW and E Romania^71,74^. A shift in river activity characteristic of lower discharge and related lower amount of precipitation again indicates the emergence of warmer and drier conditions^75,76^. The highest peaks in organic matter (LOI 550: 20–23%), peak negative δ^13^C_org_ values (− 16.2‰) as well and a peak in clay further underlie the scenario of a marked low stand (Fig. 4). A positive shift in δ^18^O of shells (to − 10.8 to − 11.2‰), and a reduction in As, Pb, Sr marks the periodic cessation of the thermal water supply (Figs. 4, 5). In this stratigraphic interval, a marked accumulation of macrocharcoal is noted (Fig. 3). In addition, it is the only interval when shallow water *Chara hispida* remains appear^77,78^. These remains are completely absent from the rest of the sequence indicating that the strikingly shallow conditions of the oligomesotrophic phase occurred only during this low stand and that higher water levels were not subsequently suitable for the organisms. The presence of the ostracod *Candona wetzleri* also confirms this scenario with water temperatures not exceeding 18 °C^79,80^.

After this short interval there is a very pronounced stepwise shift in all parameters (δ^18^O shell to negative, δ^13^C_org_ and δ^13^C_shell_ to positive, δ^15^N_org_ to negative, As, Pb, Sr increase, organic matter rapidly decreases as well as concentration of detrital elements) from ca 10.4 ka cal BP marking increased precipitation, thermal water input into the system and a rise in the lake level most likely connected to the humid conditions of the 10.3 ka event (Figs. 3, 4, 5, 6).

In the remaining oligomesotrophic lake phase up to 5.4 ka cal BP a gradual decrease in clay content, the concentration of detrital elements, reduced charcoal and shell sulfur concentrations, low organic matter content, the disappearance of shallow water gastropods and characea marks a slow gradual increase in the water table and the emergence of a deeper water carbonate-rich oligomesotrophic lake because of increased precipitation (Figs. 3, 4). The carbonate content of the lake increases reaching peak values around 6 ka cal. BP. Iterative increases in the carbonate content, shell Ca, and sediment Ca values as well as concentrations of minor carbonate nodules in the sediment are accompanied by iterative abundance increases of the thermophilus gastropod *Theodoxus prevostianus* indicating that these periods of increased carbonate input must represent increased warm water discharge into the lake, creating ideal conditions for the thriving of the mentioned warm water gastropod taxa. Elements of strictly natural origin enriched in thermal waters (As, Sr, Pb) also show a strong correlation with the mentioned sedimentological parameters and periodic negative shifts of oxygen isotopes indicating the supply of depleted waters into the lake (Figs. 3, 4, 5, 6).

Oxygen isotopes increase up to about 8 ka cal BP, δ^13^C_shell_ also, Mg/Ca ratio and thermal water sourced Pb, As, Sr remains low and constant. The abundance of the thermophilus *Th. prevostianus* reaches its all-time minima indicating relatively stable water levels and lower temperatures (Figs. 3, 4, 5, 6). Change is notable after 7 ka cal BP with oxygen isotopes gradually turning negative again. Thermal water sourced Pb, As and Sr in the shells as well as Mg/Ca ratios, and carbonate content show a stepwise increase from here marking intense thermal water input into the lake and rising water levels and temperatures (Figs. 3, 4, 5, 6.). Reduced charcoal accumulation rates in Lake Stiucii and T. Muced^69–71,74^ as well as in our records (Fig. 3) marks an overall decrease in fire activity. The gradual decrease in oxygen and carbon isotope values of nearby speleothem records in the Apuseni Mts^67,73^ as well as the high MAP values reconstructed for the nearby peatlands^69–71,74^ (Figs. 3, 4) marks the emergence of cooler and wetter conditions offering a continuous supply of water into the underground karst system and ensuring constant water input into the lake and the maintenance of relatively higher water levels (Figs. 3, 4). At 6.3–6 ka cal BP an increase in concentrations of carbonate concretions, abundance of the thermophilus *Th. prevostianus* along with a negative shift in δ^18^O_shell_ and positive shift in δ^13^C_shell_ may indicate renewed warm water input into the lake (Fig. 3). Our record also shows an increase in detrital material input (Al, Ti, Si) (Fig. 4). The coeval increase in MAP at Steregoiu^69–71^ and decreases in oxygen and carbonate isotope values of Apuseni Mts speleothems^67,73^ hint at increased availability of moisture in this interval (Figs. 3, 4).

Another charcoal peak turns up in our records between 6 and 5 ka cal BP marking renewed fire activity (Fig. 3) The abundance peaks of the eutrophic shallow water gastropod taxa *Anisus spirorbis* and the littoral habitat preferring *Succinea putris*, a marked positive peak in δ^13^N_org_ values and a rapid negative shift in δ^13^C_shell_ and positive shift in δ^18^O_shell_ as well as increasing shell S values mark the development of shallow water conditions, increased swampification under drier conditions. (Figs. 3, 4). This major low stand (LS2) is the second after the first one between 11.4 and 10.3 ka cal BP. Low lake levels and reduced river activity are documented between 5.5 and 5.3 ka cal BP in the Eastern Carpathians and the Transylvanian Plain too^75,76^. Dry peat surface conditions and general increase in fire activity was identified between 5.5 and 4.8 ka cal BP in the Romanian Carpathians^69–71,74^. Around 5.5 ka cal BP warmer/drier climatic conditions develop as seen on speleothem isotopes from the Apuseni Mountains^67,73^ and pollen-based quantitative climate reconstructions in the Gutâiului Mountains, NW Romania^69–71^ (Figs. 3, 4).

After ca. 4.5 ka cal BP there is a marked decrease in the D50 values, nitrogen isotope values, a sharp increase in the concentration of small carbonate concretions and the abundance of *Th. prevostianus* (Figs. 3, 4) accompanied by the complete disappearance of the eutrophic shallow water gastropod taxa. One of the most prominent negative δ^18^O_shell_ peaks also hallmarks the strong input of thermal water and a rapid lake level rise giving birth to the eutrophic thermal lake. The sharp decrease in charcoal concentrations in our deposits here is also notable (Figs. 3, 4). There is an abrupt rise in the lake levels of the Eastern Carpathians from 5.3 ka cal BP^81^ and the Transylvanian Plain from 5 ka cal BP too^69–71^. A marked negative shift in oxygen and carbon isotopes of the Ursilor cave speleothem here with all-time low values around 4 ka cal BP marks the emergence of cooler and wetter conditions^73^ (Figs. 3, 4). Cooler and wetter conditions associated with the 4.2 kyr event^82^ have been widely documented in the region by various records of fluvial activity, oxygen isotopes^73,75^, chironomid-based summer temperature^72^ and mire surface wetness reconstructions^83^.

From ca. 3 ka cal BP there is a marked increase in the sedimentation times to all-time high in the entire profile (44 cm/y) (Figs. 3, 4). This is accompanied by a shift to a dominance of finer grain-size classes with a marked increase in the clay content. The concentration of small carbonate concretions also decreases, δ^18^O_shell_ increases with a concurrent positive shift in carbon isotope values marking increasing temperatures, and eutrophication is also signaled by gradually increasing S values in the shell and the abundance of shallow water gastropods. It refers to the emergence of a shallow (1–2 m deep), eutrophic lake (Figs. 3, 4). This drop in the lake level might be partially attributed to the emergence of drier conditions also seen in the increase in charcoal concentrations in the sediment marking intensifying fire activity in the neighboring areas. A high peat decomposition and elevated charcoal accumulation rates at 2.7 ka cal BP at the Tăul Muced peatbog NE Romania suggest also dry climate conditions^71,74^. High fire activity and a lowering of the lake level at Ştiucii Lake, higher MAT and lower MAP inferred from the pollen records of the crater lakes of Steregoiu and P. Tiganului in NW Romania as well as high carbon and oxygen isotope values in Ursilor Cave speleothems at the Apuseni Mts, NW Romania also indicated decreasing moisture availability around 2.8 ka cal BP^67,69–71,73^ (Figs. 3, 4). This change is linked to reduced solar activity inferred at 2.8 ka from many peatlands in NW Europe^84^. Paleohydrological data show dry climate conditions during this time at a wider regional scale in Europe (Poland, Germany)^85^.

After 2 ka cal BP speleothem data from Ursilor Cave^73^ indicate a cooling of the climate with the development of wetter conditions in general hallmarked by wet surface mire conditions at Tăul Muced peatbog NE Romania^69–71^. This increase in moisture availability must have contributed to a slight rise in the lake level at Lake Pețea as seen in a stepwise decrease in the accumulation times to 26 year/cm (Fig. 4). A parallel increase in the concentration of small carbonate concretions, a slight sand input and rise in detrital elements, and a minor rise in the abundance of the thermophilus gastropod *Th. prevostianus* here may hint at the increased water discharge of the underground hot springs feeding the lake coevally with the 1.4 ka event (Figs. 3, 4). The last peak of shallow water gastropods and shell S and other isotopes marking dry conditions and a lowered lake level are dated to the period of the MCA. After that, all proxies point to the development of humid conditions and slightly increased water levels^54^.

Modern-day precipitation is highly depleted in oxygen isotopes (− 9.9‰)^60^. Thermal waters feeding the lake in the area are likewise heavily depleted (from − 12.3 to − 10.30‰, mean: − 10.9‰)^52,60^. So, a negative shift in both lacustrine carbonate and shell carbonate oxygen isotopic values concomitantly with a positive δ^13^C_shell_ shift signals reduced bioproduction due to periodic higher inputs of thermal water and/or precipitation. Extant rescued *Microcolpia parreyssii *shells had significantly higher δ^18^O_shell_ values (mean: − 10.2‰) than those of the fossil ones and plotted closer to the modern values of thermal waters and precipitation recorded in the region (Fig. 3)^60^. Highly negative δ^13^C_shell_ values (mean: − 3.6‰) indicated elevated bioproduction compared to prehistoric times. In the entire profile δ^18^O_shell_ values of *Theodoxus* and *Microcolpia* clearly overlap and show a similar upward increasing trend to those of δ^18^O_carbonate_ and regional speleothem records^67,73^. Late glacial, early- and mid-Holocene samples are heavily depleted compared to younger ones (Fig. 3). Likewise, δ^13^C_shell_ values are less depleted in the earlier oligomesotrophic part of the lake than in the eutrophic thermal water phase.

### Assessment of temporal morphological variation of *Microcolpia* and *Theodoxus prevostianus* and its potential causes in light of inferred paleohydrological changes

Three major shape traits (PCs) capture over 80% of shape variation. The most important shape trait (PC 1–68.6%) is related to the globularity of the shells (Fig. 5) along with the appearance of shoulders in case of positive values. PC 2 mainly describes shape differences of the body whorl with negative values representing globular, positive values representing flattened flanks with elongated apertural areas (Fig. 5). Finally, the third shape component (PC 3) giving 5.3% of shape variation captures minor variations in both the body whorl and the spire (Fig. 5). Shapes with negative eigenvalues preserve the apical angle of the consensus shape and there is an equal flattening of the sides of the body whorl as well as the lower part of the spire. This brings about the emergence of a strong shoulder just above the apertural area. Shapes with positive PC 3 eigenvalues are characterized by a uniform inward displacement of all outline points compared to the consensus shape creating slenderer spindle-like shapes (Fig. 5). Variations in shell globosity appear to be allometry-related, with smaller specimens being bulkier and lower-spired while larger specimens being elongated and relatively higher-spired^58^. Axial ribs and keels, originally considered to have emerged in the second step of evolution^26^, appear randomly. Carination was also recorded on thermal water melanopsid *Microcolpia daudebartii doboi* (Schréter, 1975) with elongated, high-spired, spindle-like shapes from Early Pleistocene deposits of Eger, Hungary^35^ pointing to the presence of potentially similar factors influencing the development of these structures.

**Figure 7:**
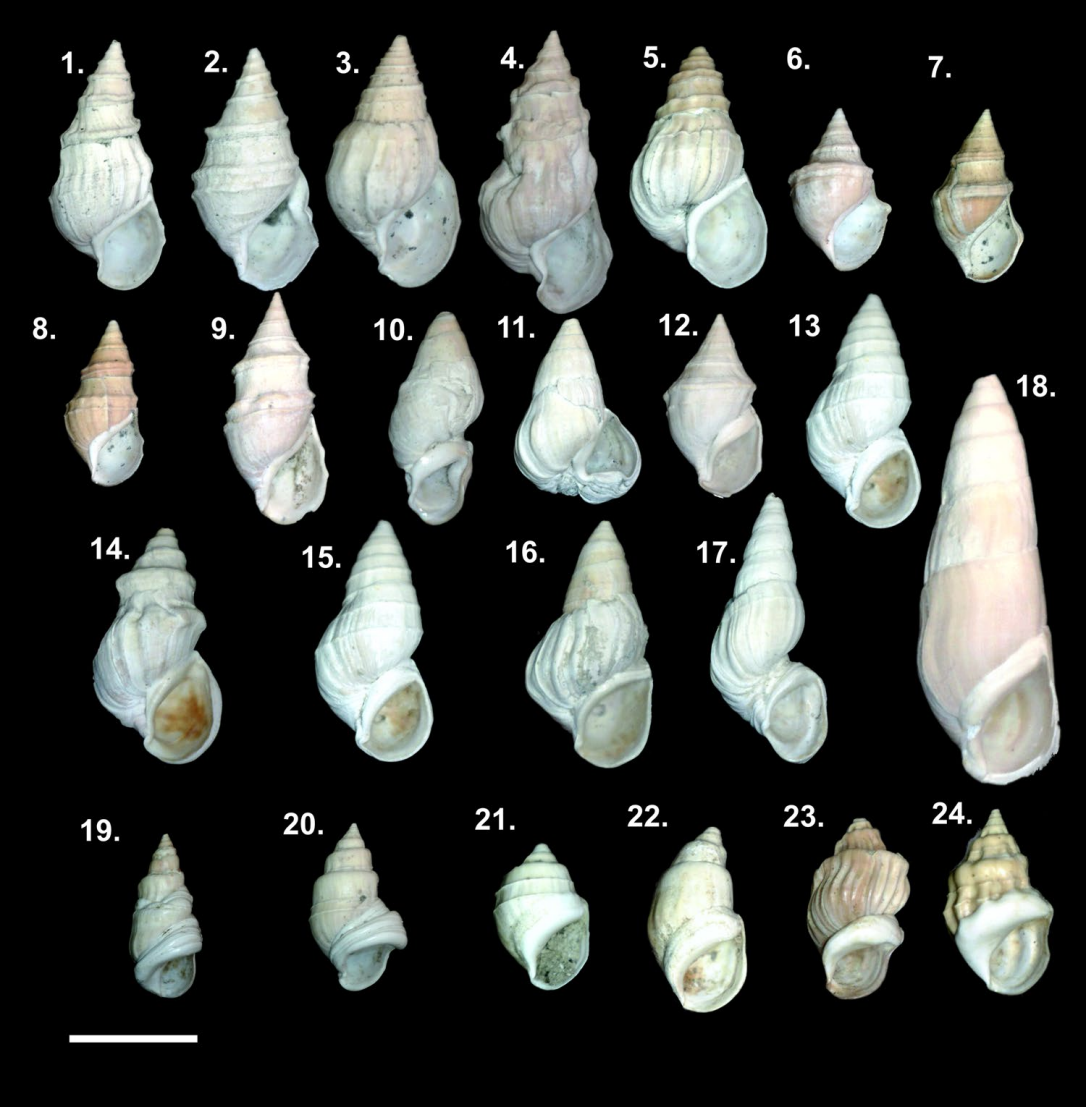
*Microcolpia daudebartii hazayi* (1–18) and *Microcolpia parreyssii* (19–24) specimens displaying abnormalities in shell growth and shape during the very early phase of the oligomesotrophic lake dated to the Late Glacial (1–8), right after the first major lake level drop at 11 and 13 ka cal BP (9–13), during the early and mid-Holocene (14–18) as well as after the second lowstand at 5.5–5ka cal BP (19–24).

Globose shells of *Microcolpia* (PC1 > 0) are restricted to GI-1 and the upper part of the last ca. 5 kys (Fig. 5). During GI-1 there is a moderate increase in shell size, aperture circumference, shell thickness and density as well as the whorl expansion rate (Fig. 6). PC2 remains negative marking the emergence of shells with more conical body whorls and spire, while PC3 remains positive very close to the consensus shape. Globosity (PC1) seems to show a similar trend recorded by parameters marking increased warm water input (negative δ^18^O_shell,_ high As, Sr, Pb in shells, high abundances of *Th. prevostianus*, carbonate concretion concentrations, positive δ^13^C_shell_) and Ca-Mg availability (high Mg/Ca in the shells) mainly driven by provision of thermal water being the major source of these elements. In this phase abnormal shells displaying thick carinae, vertical ribs with notches and well-developed shoulders appear (Figs. 2, 7). Towards the end of the Late Glacial (H0) there is a decrease in size, aperture circumference, and PC1 values marking the development of elongated shells with lower spire (increasing PC2, constant positive PC3). Shell thickness and density also decreases along with the concentration of warm water sourced elements (As, Sr, Pb) in the shells, a positive shift in δ^18^O_shell_, and a negative shift in δ^13^C_shell_.

In a major part of the Holocene oligomesotrophic lake phase PC1 remains below values of the consensus shape (PC1 = 0). There is an upward decrease in globosity along with an increase in size leading to the development of larger slender spindle-like shells (Figs. 2, 5). As seen on PC2, (PC2 ≤ $0$) shapes stay elongated with more globular sides of the body whorl close to the consensus shape. A shift to flattened flanks is notable in the upper part only. PC3 also stays relatively constant close to the consensus shape during the oligomesotrophic phase. A marked negative shift to more elongated body whorl types is noted again in the upper part. There is a reduction in size, globosity at the first major lowstand (LS1) dated between 11.7 and 10.4 ka cal BP. Shell density and thickness remains low.

Shell density and shell thickness shift markedly in a positive direction around 10.3 ka cal BP (Fig. 6). At the same time, a slight increase in the Mg/Ca ratio of *Microcolpia* shells, as well as in their arsenic and strontium contents, is observed. In addition, a negative shift in δ^18^O (0.4‰) and a parallel positive shift in δ^13^C (0.35‰) are accompanied by a smaller peak in carbonate concretions > 0.5 mm and in the abundance of thermophilic *Th. prevostianus*. This suggests that after the first major lowstand (LS1), the thermal spring supplying the lake with warm water became active again, resulting in a rise in lake level. This reactivation is the outcome of elevated moisture availability, higher precipitation and consequently elevated groundwater levels linked to the 10.3 ka event.

Conditions relatively stabilize with all parameters remaining relatively constant between about 9.8 ka and 7 ka cal BP, after which the whorl expansion rate, the Mg/Ca ratio of the shells, the arsenic, strontium and lead contents, the concentration of fine carbonate concretions increases till 6 ka cal BP (Figs. 5, 6). Shell density, shell thickness decrease gradually, shell globularity reaches its all-time low value, while shell size reaches its maximum. This period coincides with the largest lake level and increased thermal-spring activity seen in high abundance of thermophilic *Th. prevostianus*, a negative shift in δ^18^O_shell_ too. During the second lowstand phase (LS2) dated between 5.5 and 5 ka cal BP a stepwise shift in size to smaller values, increase in globularity, the flatness of the body whorl, a decrease in shell thickness and density is notable (Figs. 5, 6). The emergence of a thermal eutrophic lake phase around 5 ka cal BP brings further changes in shell shape and size traits. There is an increase in shell globularity leading to the development of pronounced shoulders, a large body whorl with flattened flanks and elongated tear-drop shaped apertures (Figs. 5, 6). Shell density and thickness also increases. In addition, abnormal shapes appear (Fig. 7). Shell size decreases while the aperture circumference increases. This type of dwarfism is characteristic in thermal water habitats^28^.

Whorl expansion rates tend to show a strong positive correlation with proxies signaling increased thermal water input (negative δ^18^O_shell_ and high As, Sr, Pb in shells, high abundances of *Th. prevostianus*, carbonate concretion concentrations) and Ca, Mg availability (Mg/Ca in the shells) (Fig. 6). Shells of *Microcolpia* are generally loosely coiled when warm water input increases and Ca slightly decreases with an increase in Mg and vice versa. The coiling ratio is negatively correlated with shell size. An expansion of the whorl rate allows for the conservation of calcium carbonate via changing the surface-to-volume ratio of the shell^86^ also seen in the decreased densities of our shells. Changes in whorl expansion also follow the same trend as the most important shape trait of globosity (PC1). Namely slender, elongated tightly coiled shells must have formed in relatively Ca-enriched environments, while the construction of thick loosely coiled globular shells with ribs, and keels is possible in Mg-rich slightly Ca deficient waters. So, this seems to be the main driving factor of the shell morphological evolution and shell density as well as the shell thickness. Plastic shell responses are not limited to shell thickening via increased carbonate but altering shell shapes too like the aspect ratio leading to the development of more compressed globular shapes with shoulders in our case, as this may not require additional investment in shell deposition just a reallocation of shell materials allowing for the development of adaptive shell forms^1,2,5–7^.

**Figure 8:**
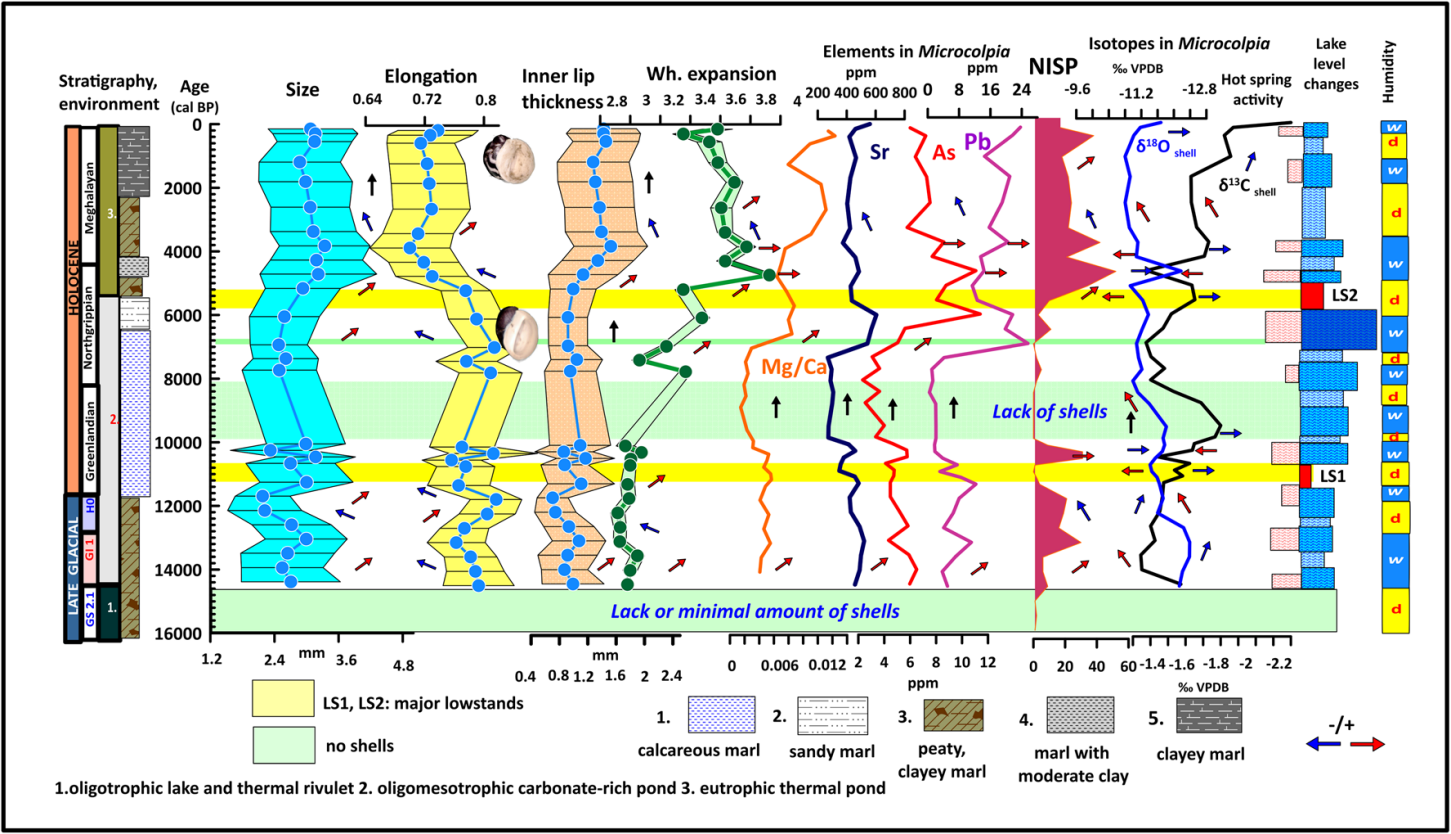
Morphological variance of *Th. prevostianus* in light of main paleohydrological proxies.

The same pattern is visible for the thermophilus *Th. prevostianus* with shell parameters showing a close correlation with Mg/Ca values of the shell and other parameters (NISP, Mg/Ca, Sr, As, Pb) indicating increased warm water input (Fig. 8). Size, lip thickness is positively correlated (r^2^: 0.75), while both have contrasting trends with shell elongation (r^2^: -0.45). A stepwise increase in size and the development of loosely coiled compressed, less elongated forms during the phase of the eutrophic thermal lake follows similar patterning to *Microcolpia* attributable to the formerly mentioned plastic shell responses to environmental change. Shells are larger, more compressed with thicker inner lips during warm water input into the lake and in general during the last eutrophic thermal water lake phase of the late Holocene. Conversely shells are smaller and highly elongated during the oligomesotrophic phase of the early and mid-Holocene apart from periods characterized by warm water pulses seen in higher Mg/Ca, Sr, As, Pb values. Globose shells are loosely coiled compared to more elongated ones, which again may reflect an adaptation to relative Ca deficiency and Mg surplus in warmer waters.

**Figure 9:**
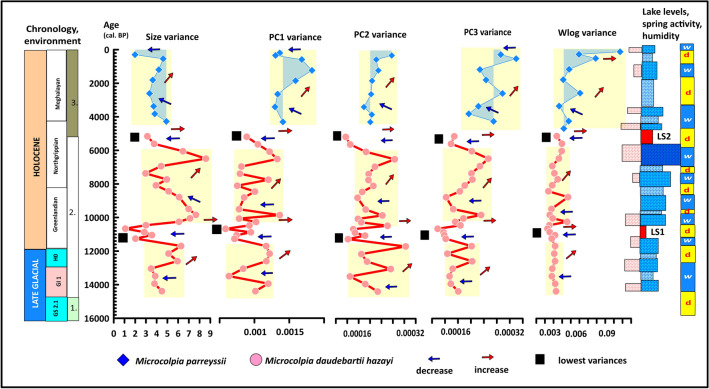
Changes in morphological disparity of *Microcolpia* with lowest variances marked at times of major lowstands and decreased Ca and Mg availability.

Morphological variance of *Microcolpia* (Fig. 9) is likewise connected to the above-mentioned processes with variances increasing at times of increased warm water input and rising lake levels. Two major intervals of the low stands (LS1, LS2) are clearly marked by extremely reduced variation in all studied parameters (size, shape, whorl expansion) besides many other small ones signaling the presence of two major bottleneck events at 11–10.5 ka cal BP and 5.5–5 ka cal BP. The succeeding shifts in major shape traits and rapid increase in variability are obvious signs of genetic drift.

## Concluding remarks, use of results in other habitat conservation strategies

Our results clearly testify how sensitively our small-size lake system responded to minor and major rapid climate change events (RCCs) of the Holocene. The hydrological changes were mainly controlled by varying input of thermal water into the lake due to recurring increased/decreased recharge of the underground shallow karst water system. Major precipitation events mainly controlled nearby erosion and detrital material input into the lake, which was especially prominent when waters were relatively shallow and succeeding extreme dry events lead to the preservation of major low stands, extreme rise in eutrophical conditions and swampification at 10.5 ka cal BP and 5–5.5 ka cal BP after the 11.4 and 5.9 ka RCCs. Our results show that melanopsids and neritids have reacted and adapted sensitively to these century-scale and millennial changes in the lake, producing extreme morphological variation.

Based on our findings endemic *Microcolpia* and *Theodoxus prevostianus* show a coordinated response to thermal water pulses, availability of Ca and Mg in shell construction and lake level changes of Lake Pețea. Periods of lower lake levels and reduced warm water input are characterized by the emergence of elongated tightly coiled shells while globular, compressed loosely coiled shells develop at times of warmer water provision and increased Mg availability. In size there is a contrasting trend. Namely globose *Th. prevostianus* shells are larger than the elongated ones. Conversely globose, compressed *Microcolpia* are generally smaller than their elongated spindle-shaped counterparts. In this sense the development of dwarf morphotypes in warmer water habitats is characteristic of Lake Pețea melanopsids alone. This type of dwarfism i.e. the reduction of shell size is lacking though in Lake Pețea neritids. Our findings also confirm the presence of various ecophenotypes of *Microcolpia* in the pond degrading our endemic species *Mi. parreyssii parreyssii* to a variant *Mi. daudebartii parreysii*.

The appearance of abnormal shells in certain parts of the profile may demonstrate periods of genetical crisis following major environmental changes—resulting in a bottleneck effect with reduced gene pool followed by genetic drift (Figs. 7, 9). The most prominent changes are notable in the lowermost records dated to the GI interval marking the emergence of the oligomesotrophic lake. Here both strong carinae as well as different degrees of shouldering and vertical thick ribs also turn up. The next part where abnormal shapes turn up is right after the first low stand (LS1) dated around 10.3 ka cal BP. The final appearance of abnormal shapes is dated to right after the second lowstand (LS2) around 5 ka cal BP.

Smooth, elongated, spindle-shaped *Mi. daudebartii hazayi*, though subordinately, is collectively present during the final 5000 years as well with the dominant shouldered smooth, ribbed morphotypes of *Mi. daudebartii parreyssii* (Fig. 2). So, thus the disappearance of other morphotypes is a very recent event which further precludes the separation of *Microcolpia parreyssii parreyssii* as separate species but rather supports its consideration as a variant of *Mi. daudebartii*.

Invertebrates, like gastropods in conservation are usually undervalued compared to vertebrates. Yet the sad story of endemic melanopsids and neritids of the thermal Lake Pețea may serve as an example of the importance of conservation of the  four remaining European habitats (Kács, Hungary; Bad Vöslau, Bad Fischenau, Austria; Pesnica, Slovenia) of highly endangered endemic thermal water gastropods (*Microcolpia* and *Theodoxus prevostianus*). Similar complex studies have not yet been carried out in these habitats, but our results suggest that they are of paramount importance.

## Material and methods

### Sampling, stratigraphy, chronology

The entire material derives from a 8.4 m deep geological profile deepened in the central part of the lake^45–47^ (Fig. 1). Block samples were taken at 0.2 m intervals. The bedrock limestone gravels, and clayey silt is overlain by ca. 2 m thick silt and organic-rich sediments (peaty, clayey silt). This is topped by ca. 4 m of calcareous marl up to a depth of 2.5 m with thin pebbly, sandy intercalations in its upper part. The final units are a 1 m organic-rich lacustrine sediment with thin silty, sandy marl intercalations overlain by 1 m of clay-rich silty marls. The temporal evolution of the lake is divided into three intervals^54^. Phase 1 represents an oligotrophic lake and thermal rivulet between 17 to 14.7 ka cal BP. Phase 2 covers the period of an oligomesotrophic carbonate-rich lake from 14.7 to ca. 5.5 ka cal BP, and phase 3 represents the stage of a Late Holocene eutrophic thermal lake till 546 y cal BP^54^ (Fig. 1).

### Sedimentology, environmental magnetism, freshwater mollusk paleoecology

For a comprehensive interpretation of the paleohydrological changes results of detailed sedimentological and geochemical analysis of lacustrine samples (grain-size, organic and carbonate content, environmental magnetism marking input of magnetic rich minerals, concentration of small carbonate concretions > 0.5 mm related to underground spring water activity, concentration of microcharcoal representing intensive vegetation fires) published previously^54^ was adopted.

The abundance of shallow water gastropods living in organic-rich, alkaline waters or near-shore areas was employed to indicate the potential lower levels of the pond, as supported by other sedimentological and geochemical data^45–47^. One must bear in mind that the profile was deepened in the central part of the lake, so the abundant presence and high values of these shallow water markers in our sequence must truly mark major drops in the lake level. Abundance variations of the thermophilus endemic gastropod *Theodoxus prevostianus* (C. Pfeiffer 1828) indicate water temperatures reaching and or exceeding 16–23 °C^36,37,44–47^ signaling thermal water pulses into the lake and rising water temperatures.

### Isotope, elemental composition of sediment and gastropod shells

The concentration of selected elements in lacustrine deposits marking detrital material input (Al, Ti, Si, mineralogy), water temperature (Mg, Ca) or productivity (P) changes, as well as selected elements recorded in shells reflecting water temperature changes (Mg/Ca ratio, Sr), increased thermal water input into the lake (As, Pb, Sr), swampification due to rotting of organic matter (S), were determined as part of this study. Shell and sediment samples were pulverized, and powder pellets were analyzed using a Horiba energy dispersive XRF XGT-1000WR. The mineralogical composition of the sediment was determined by a Rigaku Ultima IV XRD.

Use of stable isotopes recorded in sediments and lacustrine organisms’ shells is an essential part of paleolimnological investigations. Changes in oxygen isotopes of shells and lacustrine carbonate are important proxies for paleoclimate as they reflect changes in the source of water to the lake and/or temperature, precipitation/evaporation ratio^61–63^. Due to the extremely small size of the lake and its dominant source of thermal groundwater, the isotopic composition must reflect changes in spring activity and rainwater input from precipitation.

Changes in carbon isotopes recorded in the shell and sediment organic matter (SOM) reflect the trophic status of the lake^61–63^. Nitrogen isotopes are more difficult to interpret but generally carry information on the nitrogen cycling in the lake with negative values attributable to higher number of algae while positive values may hint to drier conditions^61–63^.

Shells of extant rescued and fossil *Microcolpia* and fossil *Th. prevostianus* were analyzed for isotopic (δ^18^O_shell_, δ^13^C_shell_) composition in addition to lacustrine calcite and sediment organic matter (SOM) (δ^18^O_carbonate_, δ^13^C_carbonate_, δ^13^C_org_, δ^15^N_org_). Conventional carbon and oxygen stable isotope analyses were carried out on carbonate powders with an automated GASBENCH II sample preparation device (phosphoric acid digestion at 72 °C) attached to a Thermo Finnigan DeltaV Plus continuous-flow isotope ratio mass spectrometer (IRMS)^87^. Results are expressed as δ^18^O and δ^13^C values relative to Vienna Pee-Dee Belemnite (VPDB). The precision of the measurements is ≤ 0.08‰ for δ^13^C and ≤ 0.1‰ for δ^18^O.

Stable isotopes of SOM (δ^13^C_org_, δ^15^N_org_) were measured on a Thermo Scientific™ EA IsoLink™ IRMS System for CNSOH known as Flash EA, which is an enhanced elemental analyzer for nitrogen and carbon isotope analyses as well^88,89^. This is based on a flash dynamic combustion method, which produces complete combustion of the sample within a high temperature oxidation reactor, which allows the complete conversion of all samples, to elemental gases (N_2_ and CO_2_). The separation of dedicated gases is carried out on chromatography column, after gas separation, the gases are introduced to the mass spectrometer by helium carrier gas. The Flash EA 1112 is connected to an IRMS system (Thermo Finnigan DeltaPLUS XP continuous-flow isotope ratio mass spectrometer) for the accurate determination of nitrogen and carbon isotope ratios. Samples (0.25 ± 0.04 mg) were weighed into ultra clean aluminum capsules for carbon analysis, then dropped into the autosampler of the elemental analyzer. Results are expressed in conventional delta notation as follows, δ (%) = (Rsample/Rreference − 1) * 1000, where R is the ^15^N/^14^N or ^13^C/^12^C ratio of the sample or reference standard and the δ^15^N and δ^13^C values are relative to AIR and VPDB international reference points, respectively. Every sample was measured at least three times for each stable isotope and standard deviations of individual δ^15^N and δ^13^C measurements were ± 0.1‰ and ± 0.2‰, respectively.

### Morphometric analysis of *Microcolpia* and *Theodoxus* gastropod shells

Morphological variation of the *Microcolpia* types as well as *Theodoxus prevostianus* without reference to any previously described taxa was assessed using shell outline analysis (EFA- elliptic Fourier shape analysis) on a mass database of 100 specimens per sample totaling around 3500 studied and quantified specimens^58^. The matrix of harmonic components was subjected to PCA and the received PCs were taken as the most prominent shape traits. In addition, shell size, whorl expansion rate and traditional length and angle-based morphometric parameters have been recorded enabling comparison with recent specimens^42,59^, where only such parameters were available. Besides the determination of major shape traits (PCs), quantified parameters included proportional density of the shells (greyscale value) and shell thickness (mm) derived from micro-CT analyses^90^. 30 specimens from each sample were scanned at full rotation at a pixel size of 85 microns at a resolution of 1500 × 1500 pixels with a voltage of 90 kV and a current of 230 μ using a Bruker Skyscan 2211 Nano-CT scanner. For each specimen, all reconstructions were performed in ORS Dragonfly using the same reconstruction parameters (beam hardening, ring artifact reduction, attenuation coefficients) to ensure that the scans were directly comparable. Density was calculated after segmentation and binary thresholding of the image stacks relying on the received grayscale histograms as proxy for relative shell density. As comparability between the scans of individuals and a pack of individuals per sample was required there was no need for the determination of absolute density (Hounsfield unit values). The range of the greyscale histogram used for segmentation was the same for all specimens (60–255). 3D analysis was performed in software packages Dragonfly and Morphodig. After visualization and segmentation of volumes, thresholded surfaces were created and scalar thickness calculated in Morphodig.

### Supplementary Information

Supplementary Figure S1.

Supplementary Legends.

## Data Availability

Data is available upon request from the corresponding author.
