# Supplementary figures and images for: Coordinated response of endemic gastropods to Late Glacial and Holocene climate-driven paleohydrological changes in a small thermal pond of Central Europe
Source: Sci Rep. 2024 Apr 24;14:9419. doi: 10.1038/s41598-024-60185-5 (PMC11043081; doi:10.1038/s41598-024-60185-5)

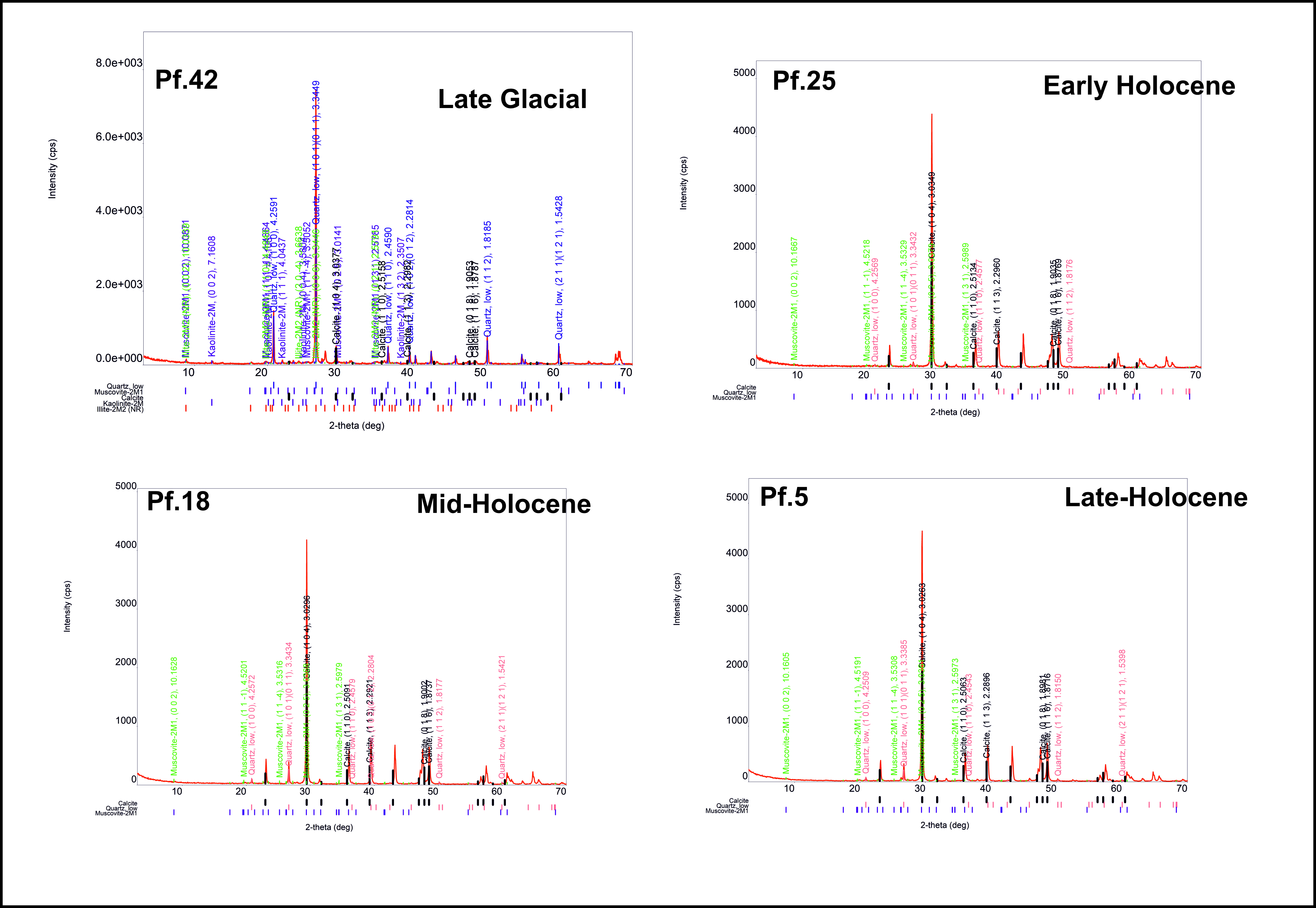

Supplement: Supplementary file 1 — Supplementary Figure S1. [file 41598_2024_60185_MOESM1_ESM.tif]
