# Supplementary material for: Coordinated response of endemic gastropods to Late Glacial and Holocene climate-driven paleohydrological changes in a small thermal pond of Central Europe
Source: Sci Rep. 2024 Apr 24;14:9419. doi: 10.1038/s41598-024-60185-5 (PMC11043081; doi:10.1038/s41598-024-60185-5)
Supplement: Supplementary file 2 — Supplementary Legends. [file 41598_2024_60185_MOESM2_ESM.docx]

SFig.1 Mineralogical composition of the Late Glacial oligotrophic and Early and Mid-Holocene carbonate-rich oligomesotrophic and Late Holocene eutrophic thermal lake deposits
